# Women with mobility disabilities’ experiences of maternity care during pregnancy, labour and puerperium in Eswatini

**DOI:** 10.4102/hsag.v27i0.1861

**Published:** 2022-11-30

**Authors:** Fortunate Magagula, Annie Temane, Anna G.W. Nolte

**Affiliations:** 1 Department of Nursing, Faculty of Health Sciences, University of Johannesburg, Johannesburg, South Africa

**Keywords:** women with mobility disabilities, experiences, maternity care, pregnancy, labour, puerperium

## Abstract

**Background:**

People living with disabilities are often women and the elderly and those from low-income families. There is paucity in research on women with mobility disabilities’ experiences of maternity care during pregnancy, labour and puerperium in Eswatini.

**Aim:**

To explore and describe women with mobility disabilities’ experiences of maternity care during pregnancy, labour and puerperium in Eswatini.

**Setting:**

The study was conducted in the Hhohho and Manzini regions in Eswatini, between March 2019 and July 2019.

**Methods:**

A qualitative, exploratory, descriptive and contextual research design was followed. Purposive sampling and snowballing techniques were used to sample women with mobility disabilities to participate in the research. Individual interviews were conducted, and Giorgi’s method of data analysis was used.

**Results:**

Women with mobility disabilities experienced midwives as being brutal, unsupportive and judgemental. In addition, participants reported several maternity care challenges related to a lack of protocol and infrastructure, and they needed to be cared for by experienced midwives during pregnancy, labour and puerperium.

**Conclusion:**

Women with mobility disabilities experienced various challenges during pregnancy, labour and puerperium in Eswatini. There is a need to develop guidelines to facilitate support and holistic maternity care for these women.

**Contribution:**

The study contributed to the scientific knowledge of women with mobility disabilities’ experiences of maternity care during pregnancy, labour and puerperium.

## Introduction

The World Health Organization (WHO), using the International Classification of Function and Health, defines ‘disability’ as an umbrella term covering impairments, activity limitations and participation restrictions (Disabled World 2019:1). Furthermore, the WHO defines an ‘impairment’ as a problem in bodily function or structure; an ‘activity limitation’ as a difficulty encountered by an individual in executing a task or action; and ‘participation restriction’ as a problem experienced by an individual in various life situations. Therefore, disability is not merely a health problem but a complex phenomenon, showing the interaction of the person’s body and the environment in which they live (Disabled World 2019:1). This implies that measures to promote the health and lives of people with disabilities require interventions that will eliminate environmental or societal impediments.

Redshaw et al. (2013:2) indicate that there is increasing evidence that suggests people with disabilities – in general – have poorer levels of health, lower educational achievements and a higher rate of unemployment than their counterparts without disabilities. Many women with physical disabilities have reported barriers in accessing healthcare providers and facilities. Research further indicates that there are challenges with infrastructure and resources to accommodate the needs of women with mobility disabilities (Nguyen et al. 2019:191; Sonalkar et al. 2020:138). Ganle et al. (2016:8) report that women with disabilities face significant challenges in accessing skilled maternity care, as they often cannot reach the health facility because of their disabilities. In support, the WHO (2021:1) claims that people with disabilities do not receive the health services they need as they cannot afford health care. These individuals are also twice as likely to find healthcare providers’ skills inadequate.

Studies show that women with mobility disabilities face challenges during their pregnancy, labour and puerperium, including prejudice and being viewed as people who cannot take part in sexual and reproductive activities (Mac-Seing et al. 2020:279; Morrison et al. 2014:1133). In addition, Morrison et al. (2014:1133) mentioned that women with disabilities are thought not to be sexually active and less likely to have children than those who do not have a disability.

In addition to the aforementioned challenges, women with disabilities encounter negative attitudes, such as pity, uneasiness, being overly sympathetic and guilt from family members and healthcare professionals regarding their physical and financial constraints (Tarasoff 2015:92). The lack of knowledge regarding these constraints could also lead to women with mobility disabilities being stigmatised or discriminated against. Walsh-Gallagher et al. (2013:298) report that healthcare professionals tend to view women with disabilities as liabilities and regard them as high risk; they often exclude them from the individualised plan of care, which leads to an increase in these women’s fears about their maternity care. In addition, Shapiro (2019:2) claims that healthcare professionals are seldom trained to provide reproductive care to women with disabilities. These challenges often result in health disparities and prevent women with mobility disabilities from receiving optimal maternity care. Therefore, Redshaw et al. (2013:2) argue that healthcare professionals need to focus more on women’s abilities than their disabilities during pregnancy, labour and puerperium.

According to the global population report (United Nations 2019:1), more than 1 billion people have some form of disability. Eswatini is classified as a middle-income country; it is situated in the Southern African region and measures 17 000 square kilometres, with a population of 1 093 238. Of the population, 76.2% resides in rural areas (833 472) and 23.8% (259 766) in urban areas (Swaziland, Central Statistics Office 2019a). The economy is largely agricultural, as most industries manufacture agricultural products (Swaziland, Central Statistics Office 2018). Of the Eswatini population, 146 554 (13%) live with disabilities, with most being women (87 258 [16%]).

Fifteen per cent (125 545) of people with disabilities live in rural areas, and 85% of the population with disabilities are unemployed (Swaziland, Central Statistics Office 2019a), which means most of these individuals are economically disadvantaged. From the researchers’ observation, the population with disabilities, including women with disabilities, receive some form of stipend every 3 months.

Furthermore, according to the Eswatini Swaziland, Central Statistics Office (2019b), 26.5% of people with disabilities have a mobility (walking) disability, with 63.5% of these being women. Yet there is a lack of evidence about the maternity care these women with mobility disabilities receive in Eswatini. There is also little documented on these women’s experiences of maternity care. Thus, the research question that arose from the problem statement was: ‘what are women with mobility disabilities’ experiences of maternity care during pregnancy, labour and puerperium in the Hhohho and Manzini regions in Eswatini?’ The study thus aimed to explore and describe women with mobility disabilities’ experiences of maternity care during pregnancy, labour and puerperium in Eswatini.

## Research design

A qualitative, exploratory (De Vos et al. 2013:64), descriptive (Grove, Gray & Burns 2015:69) and contextual (Creswell & Creswell 2018:300) research design was applied in this study to gain insight and an understanding of the research phenomenon. The phenomenon under study was women with mobility disabilities’ experiences of maternity care during pregnancy, labour and puerperium, and the researcher’s interpretation of these experiences.

### Research setting

The setting of the study was the Hhohho and Manzini regions of Eswatini, which have the highest concentration of the population, with Manzini having the largest share of population at 32.6% and Hhohho at 29.3% (Swaziland, Central Statistics Office 2019:3). The fertility rate of Eswatini stands at 3.3 births per 1000 women (United Nations Population Fund [UNFPA] 2019:9), with highly skilled antenatal care at 98.5%, skilled birth attendance at 88% and institutional deliveries at 87.7%; however, the maternal mortality rate stands at 452 per 100 000 live births (UNFPA 2019:10). The researcher collected data at the site where participants experienced the phenomenon, within the context in which they were comfortable to be interviewed.

### Population and sampling

The study population was all women in Eswatini with mobility disabilities residing in the Hhohho and Manzini regions. The women with mobility disabilities were recruited through purposive sampling and network sampling. The Federation of Women with Disabilities in Swaziland (FODSWA) coordinator identified the women with mobility disabilities who were potential participants. The information letter was provided to the FODSWA coordinator to outline the aim, method of study and time frames of the interviews. The researcher signed a confidentiality agreement with FODSWA coordinator. The FODSWA coordinator acted as a gatekeeper and assisted with contacting the participants. After the first contact, the FODSWA coordinator contacted the researcher to indicate the women with mobility disabilities who were interested in participating in the study. Thereafter, the researcher telephonically contacted the participants to inform them about the study; most of whom were willing to participate in the study. The inclusion criteria were woman with a mobility disability; older than 18 years; who had given birth to a baby (alive or dead), irrespective of whether they came from a rural or urban area; who had been attended to in a health facility; and who were willing to participate in the study. The exclusion criteria were women with mobility disabilities who were less than 18 years of age, who had not had experienced giving birth and who resided outside of the Manzini and Hhohho regions. This was followed by network sampling to solicit assistance from earlier recruited participants to offer referrals of additional informants to participate in the study, as it became difficult to locate more participants. Twelve women with mobility disabilities participated in the study. Seven were from Manzini and five from Hhohho region (refer to Table 1). Eight were interviewed in their homes and four at their social gathering and regular meeting places for handwork.

**TABLE 1:** Demographics of the participants.

(P)	Region	Age (years)	Parity	Stage of maternity experience	Last pregnancy experience
1	Manzini	36	2	Pregnancy, labour and puerperium	5 years 2 months
2	Manzini	27	1	Pregnancy, labour and puerperium	6 years
3	Manzini	44	4	Pregnancy, labour and puerperium	5 years
4	Hhohho	32	1	Labour and puerperium	7 years 6 months
5	Manzini	29	2	Pregnancy, labour and puerperium	3 years
6	Hhohho	28	1	Pregnancy, labour and puerperium	4 years 6 months
7	Manzini	32	1	Labour and puerperium	4 years
8	Manzini	31	2	Pregnancy, labour and puerperium	5 years 2 months
9	Manzini	29	3	Pregnancy, labour and puerperium	2 years 6 months
10	Hhohho	40	4	Pregnancy and puerperium	3 years
11	Hhohho	39	3	Labour and puerperium	5 years
12	Hhohho	45	1	Pregnancy, labour and puerperium	8 years

*Source:* Magagula, F.N., 2021, ‘Guidelines of maternity care of women with mobility disabilities in the Hhohho and Manzini regions: Eswatini’, Unpublished doctoral thesis, University of Johannesburg, Doornfontein

The researcher opted for purposive sampling because she had prior knowledge about the population and deliberately chose the participants who would best contribute to the study (Polit & Beck 2018:291). In addition, in order to avoid being biased, the researcher employed bracketing to identify and suspend her preconceived beliefs and opinions about the phenomena under study (Polit & Beck 2018:276). The FODSWA identified women with mobility disabilities who could be potential participants. This was followed by network sampling to solicit assistance from earlier recruited participants to offer referrals of additional informants to participate in the study (Polit & Beck 2018:291). The researcher chose this sampling method because it was challenging to locate potential participants (De Vos et al. 2013:393).

### Data collection

The researcher conducted individual interviews to collect data (De Vos et al. 2013:347). The researcher holds a Master’s degree in Advanced Midwifery and Neonatal; she developed skills of interviewing during her master’s studies. After recruiting women with mobility disabilities and obtaining their permission to participate in the study, the researcher set up appointments with them for the interviews. The participants decided on the most suitable and convenient time and place for them to be interviewed; this was either in their homes or at their social gathering and regular meeting places. Permission was sought from the participants to record the interview; an audio recorder was used to record their responses, and field notes were captured throughout the interview. The central question posed to participants was: ‘what are your experiences of maternity care you received during pregnancy, labour and after the birth of the baby?’ The researcher used facilitative communication skills such as probing, exploring, clarifying and summarising during the individual interviews. Probing enabled the researcher to follow up on information that had arisen by asking specific questions and encouraging the participant to provide details and clarifications (Taylor, Bogdan & DeVault 2016:123).

The individual interviews were conducted in a language the participants were comfortable with, which was Siswati (vernacular). This was followed by back-to-back translation to English during transcription, which was resumed immediately after the interviews. The duration of the interview was 30–45 min. The interviews were conducted from March 2019 to July 2019, because of which it was difficult to locate the participants who met the sample criteria as Lincoln and Guba’s model (1985). The central question was piloted with the first participant before the subsequent interviews were conducted, allowing the researcher to refine the question should it be deemed unclear (De Vos et al. 2013:395) for the participants. The researcher did not discard the information from the pilot participant because the central question proved to be without any flaws, and it produced the intended responses and results. The researcher conducted interviews until the data became redundant and repetitive, reflecting that saturation had been reached (Polit & Beck 2018:103). The researcher recorded field notes after each individual interview.

### Data analysis

Before data analysis commenced, the researcher organised the data in computer files, after having them transcribed, and translated them into the narrative form. Data from each participant were coded and stored in the relevant file and kept in a safe place; only the researcher could access the information. Backup copies were made of all the data, and the master copies were stored in a safe to which only the researcher had access.

Data collection and analysis occurred concurrently. The researcher was guided by Giorgi’s (1985) five-step method of data analysis (Giorgi 2012:3–12). This entailed the researcher reading all the transcribed data and the entire ‘naïve description’ provided by the participants during the interviews. The demarcation of ‘meaning units’ within narratives followed. In addition, the researcher marked where meaning shifts occurred and transformed meaning units into descriptive expressions. The researcher laid out the general structure of women with mobility disabilities’ experiences. Moreover, an independent coder was provided with the raw data (after signing a confidentiality agreement) to analyse the findings. The researcher and the independent coder had a consensus discussion about the analysed data and agreed on the final themes and categories.

### Ethical considerations

Ethical clearance to conduct this study was obtained from the University of Johannesburg Faculty of Health Sciences Higher Degrees Committee (ref. no. HDC-01-50-2018), University of Johannesburg Faculty of Health Research Ethics Committee (ref. no. REC-01-82-2018) and the Eswatini National Health Research Review Board (ref. no. NHRRB982/2018).

In addition, the researcher applied the ethical principles as described by Dhai and McQuoid-Mason (2011:14–15) throughout the research process. These principles included autonomy, informed consent, confidentiality, nonmaleficence, beneficence and justice.

Autonomy relates to respect for individuals’ autonomous choices and participants’ decision-making. The principle considers self-determination based on informed consent and respecting confidentiality (Dhai & McQuoid-Mason 2011:14). The researcher kept to the principle of autonomy by affording the participants the right to choose to participate in the study (Grove et al. 2015:98). The participants were provided with an information letter written in Siswati and English to inform them of the aim of the study. Informed consent was then given by the participants to take part in the study voluntarily. Their agreement was affirmed by signing a voluntary informed consent form (Creswell & Creswell 2018:173), without any coercion, as there was no prior relationship between the researcher and the participants. The researcher met each of the participants the first time for signing of the consent form and the second time when the interview occurred.

The researcher upheld respect for participants’ privacy and protected the anonymity of individuals, roles and incidents in the project by disassociating the names from responses during the coding and recording process (Creswell & Creswell 2018:176). A confidentiality agreement was signed between the researcher and the independent coder to safeguard the data that were shared between them. According to Grove et al. (2015:107), the researcher’s function is to manage and safeguard all collected data and ensure the information is kept private from others and cannot be shared without the participants’ authorisation.

According to the principles of nonmaleficence and beneficence, researchers must avoid harm as much as possible and promote benefits for the participants in their study. Creswell and Creswell (2018:175) indicate that researchers need to guard against the possibility of harmful, intimate information being disclosed during data collection processes. Dhai and McQuoid-Mason (2011:15) also state that beneficence means doing good for others and promoting the interests and well-being of others; this should be taken into consideration in all research to safeguard participants’ welfare. In this study, the participants’ safety was ensured by conducting the interviews in a familiar, private environment where they felt free and safe from harm. Each participant was interviewed alone within a closed, quiet and secured place, with a ‘do not disturb’ note at the door. Finally, the principle of justice states that human subjects should be treated fairly in terms of the benefits and the risks of research (Grove et al. 2015:98). All the participants were thus treated with respect.

### Measures of trustworthiness

The research was informed by Guba’s (1985) model in relation to credibility, transferability, dependability and confirmability. The researcher ensured the credibility of the study by triangulating different data sources and examining evidence from these sources (Creswell & Creswell 2018:325). The evidence was used to build a coherent justification of themes through the process of induction, whereby the researcher converged the study findings with the existing literature. Member-checking was also used, and the researcher shared the final report with participants so they could assess its accuracy. The researcher had to go back to the setting where the interviews had occurred to share the final transcripts with the participants. The researcher also kept a reflexive journal and ensured bracketing by omitting any perceptions from her past experiences that were likely to influence her interpretation of the research findings.

Through prolonged engagement in the field, the researcher ensured the credibility of the findings (Polit & Beck 2018:417). Peer debriefing (Creswell & Creswell 2018:325) was also promoted through comprehensive research supervision by the researcher’s supervisors, who are experienced professors in the research field from the University of Johannesburg. Moreover, an external auditor was used (Polit & Beck 2018:417), an external examiner, at the conclusion of the study. The study was also presented on the 13th May 2021, at a Virtual University of Johannesburg Annual Research Forum and Post-Doctoral Forum, Johannesburg, South Africa.

Transferability is the ability to extend the findings of one’s study to comparable environments or participants (Pitney et al. 2020:55). The researcher ensured the study’s transferability by providing a richly documented account and an in-depth description of all aspects and processes of the study protocol. This account included the study’s background and rationale; problem statement; purpose and objectives; paradigm, which is the philosophical orientation of the study; and methodology, including data collection and analysis procedures. Furthermore, data saturation also confirmed transferability (Polit & Beck 2018:417).

Dependability in a study is evident when other researchers are able to follow the researcher’s decision trail (Leavy 2020:1096). The researcher ensured dependability by densely describing the research process (Creswell & Creswell 2018:325; Grove et al. 2015:392) so that other researchers can follow similar steps of the same research methodology in conducting similar studies. The triangulation of data collection methods, field notes, individual interviews and the use of a pilot study further ensured dependability. In addition, the researcher checked the research transcripts repeatedly and listened to the audio recordings several times to develop a dependable audit. The engagement of an independent coder further ensured the dependability of the findings.

Holloway and Galvin (2017:309) state that confirmability occurs when the research is judged by the way in which the findings and conclusions achieve their aim and are not the result of the researcher’s prior assumptions and preconceptions. The researcher ensured this by remaining true to the research process and not compromising the research process in any way, through bracketing (Grove et al. 2015:501). In addition, the researcher engaged an independent coder and provided a chain of evidence of the entire research process to enable an audit. Therefore, all forms of collected data were recorded, including raw data, reflexive journal (Creswell & Creswell 2018:301), notes and transcriptions.

## Results

Twelve women with mobility disabilities from Manzini and Hhohho regions who received maternity care during their pregnancy, labour and delivery and puerperium participated in the study. Their ages varied between 27 and 45 years, and their parity was from 1 to 4. The sample size was determined by repetitions of key statements about the study phenomenon during data collection (Polit & Beck 2018:103). The representation of the demographics of the participants is shown in Table 1. The reported excerpts are labelled with a (P), followed by a corresponding number to connect the participant with recorded statements (e.g. P1; consult Table 1).

Five themes and categories emerged from the data analysis. Table 2 represents the summary of the themes of women with mobility disabilities’ experiences of maternity care during pregnancy, labour and puerperium in Eswatini.

**TABLE 2:** Themes and categories of the experiences of women with mobilities in regards of maternity care during pregnancy, labour and puerperium.

Themes	Categories
1. Experience midwives as being unprofessional	Women with mobility disabilities reported midwives acted in an unprofessional and unempathetic manner.
2. Experience being victimised by midwives	Women with mobility disabilities reported being emotionally and physically abused by midwives.
3. Experience a lack of support during pregnancy, labour and puerperium	Women with mobility disabilities experienced a lack of support from midwives. Women with mobility disabilities reported being denied support from significant others.
4. Experience challenges before and after giving birth related to a lack of protocols, equipment and infrastructure	Women with mobility disabilities experienced challenges in relation to protocols in their maternity care related to: A lack of a designated queue for women with mobility disabilities. Expectations from midwives to walk around during latent phase of labour. Women with mobility disabilities reported challenges with a lack of equipment and infrastructure, which include: A lack of transport to attend weekly antenatal care appointments during the third trimester. A lack of accessible toilets and ramps in the health facility. A lack of equipment: examination, labour and delivery beds.
5. Experience the need for support from midwives and family	Women with mobility disabilities reported a need for support from trained midwives.

*Source:* Magagula, F.N., 2021, ‘Guidelines of maternity care of women with mobility disabilities in the Hhohho and Manzini regions: Eswatini’, Unpublished doctoral thesis, University of Johannesburg, Doornfontein

### Theme 1: Experience midwives as being unprofessional

Women with mobility disabilities reported that midwives were unprofessional and unempathetic in the manner in which they cared for them. Some women reported that midwives went to the extreme of abusing them physically:

‘Even if you had to deliver, they do not assist, as you need to hold somewhere to gather your strength to push the baby out, yet you need to get the baby out one way or the other. The nurse would tell you to hold your thighs yet I couldn’t even lift my leg cause even my other arm is disabled. They hit me hard on the thighs, thinking I’m being spiteful; they couldn’t see that I am not able to, and I needed their support.’ (P5)

‘There is the problem of nurses shouting at the women, which they sometimes do even for normal women, but it seems they overdo it on women with disability. It’s like you’ve committed a sin, it’s like we are not expected to have children because we [*women with disabilities*] are reprimanded a lot, as if we have disgraced ourselves by becoming pregnant.’ (P6)

### Theme 2: Experience being victimised by midwives

The women with mobility disabilities reported that the remarks, questions, attitudes and treatment they received from the midwives made them feel emotionally abused. Some of the women felt violated in the manner in which midwives communicated with them by asking them how they fell pregnant:

‘When I got onto the bed, there was the challenge of opening my legs for examination because they cannot open on their own due to my disability; I have to use my hands and open. The nurses would become very sarcastic and said, “How did you open them when you had sex with your man?” I felt belittled and shy.’ (P9)

‘I was well cared for in our nearest clinic [*chuckles*] … though I was a bit disturbed when they did the blood tests; the results were not well communicated. I was hurt when they disclosed the results … they said that I was not supposed to even have been in the position of having a sexually transmitted infection, if I had thought first about my disability and not had sex and got pregnant in the first place.’ (P8)

‘I was six months pregnant when I went to the clinic for antenatal care. I told the nurse that I had come to confirm if I was really pregnant. The nurse asked me, “Are you also having sex?” I was perplexed by such a question … The nurse went ahead and examined me, wrote on the card, and she said, “Who impregnated you?” I kept quiet because I didn’t like the way she was talking to me; I was no longer at ease.’ (P2)

‘When I went for antenatal care, I was asked by the nurse why I became pregnant in my condition, and that will I be able to take care of the baby … It didn’t go down well with me, cause it meant they thought that I’m not supposed to have a child in my condition.’ (P1)

### Theme 3: Experience a lack of support during pregnancy, labour and puerperium

The participants reported feeling helpless as the midwives did not support them or offer them any assistance, saying it was not their job to do so, yet even their significant others were not allowed into the labour ward. They shared that the midwives did not want to understand, were not empathetic, were impatient and were unfriendly towards them:

‘The problem got complicated when I had to climb up the bed, because even those who had accompanied me were not allowed into the labour ward. The problem, you see, was when they would say … “hold here and balance” … I couldn’t, because almost all of the other half of my body is nonfunctional … I needed support to hold wherever and maintain balance, but in my case there was no support … I only got it when eventually they could see that I couldn’t help myself at all … what ended up happening was some of the sutures from the incision were torn … cause as I was struggling with putting on the leg brace, trying to walk with it ….’ (P1)

‘[…*E*]ven when you want to get out of the bed after examination, you must see for yourself how you get down, no one cares to assist, or at least offer you a stand to step on for you to get down ….’ (P5)

### Theme 4: Experience challenges before and after giving birth related to a lack of protocols, equipment and infrastructure

Women with mobility disabilities experienced challenges with protocols, equipment and infrastructure that were not suitable to accommodate their disability. They said:

‘The queue can be very long sometimes. There is not even a provision for us who are disabled that they may start with us, cause there are even no resting chairs whilst waiting for your turn. There is not even something to lean on, yet we can’t stand properly; we don’t have balance … you wait in that condition until it is your turn … you are supposed to hold onto your number … if you are number 20, you’ll be number 20 [*emphasising the queuing*].’ (P5)

‘I went to the hospital, they examined me and said the baby is still far, I must go back home and come back if the pain gets stronger. I went and came back, they said the baby is still far I must walk around the hospital … I was walking with the crutch, it was very difficult that is why I would roll down on the floor when a strong contraction came cause I didn’t know what to do.’ (P7)

In addition, the women with mobility disabilities reported challenges from a lack of equipment and infrastructure. One of the women with mobility disabilities explained:

‘[…*T*]he toilets are not user-friendly for us, people with mobility disability … when the toilet is too low I can’t sit on it and even [*to*] stand up, it becomes difficult … the toilets are even small; they do not accommodate the way we sit, cause as for me I have to straighten my leg when sitting on the toilet … so with these toilets, you have to open the door so your leg can be free, you see … I can’t feel free.’ (P1)

Another reported:

‘The beds are too high; it can help if they can be a bit lower for them to be normal for us … they should be able to assist when they see that a person is disabled. Like in my case, they ended up putting me on a stretcher and rolled me onto the bed. They must also not look down upon us because we are disabled; they should assist us.’ (P3)

One added:

‘The public transport drivers do not want us, the time, especially if it’s the rush hour. When you finish at the clinic during the rush hour, sometimes they would leave you and not pick you up at the bus stop, cause no one wants to sacrifice and say, “Leave me and pick this one on a wheelchair.”’ (P10)

### Theme 5: Experience the need for support from midwives

Women with mobility disabilities expressed their need for trained midwives who are equipped with knowledge and skills to support them and offer them adequate maternity care. They reported:

‘Nurses require training so they can know how to take care of us women with disability. They need to ask us how we feel because we [*people with disabilities*] are scared … what I can say is that the nurses need to be specifically trained at college level on how to take care of us women with disability and how to deliver our babies ….’ (P4)

‘If these nurses could be trained to be patient and assist where required.’ (P10)

‘It’s just that the young nurses need to be trained on taking care of people with disability. Because some of the older nurses are able to even not make us queue; they have the love for us; they don’t discriminate. Though others do not care, they just pass by and ignore us.’ (p. 11)

## Discussion

Childbirth is a special experience that requires a personal connection between the midwife and the woman giving birth, characterised by successful communication and respect (Hallam et al. 2016:183). The themes identified in the study indicated that women with mobility disabilities experienced challenges with maternity care during pregnancy, labour and puerperium; the lack of infrastructure and protocols; and a lack of support. The themes also highlighted a need for support from qualified midwives.

One of the themes centred on participants’ experiences of midwives being brutal and unsupportive. Midwives reportedly lacked professionalism in their provision of maternity care for women with mobility disabilities. Professionalism in midwifery is displayed through the midwife’s character and self-leadership, as well as adherence to ethical codes under the direction of professional scientism (theoretical knowledge and professional skills), professional communication, sympathetic and trust-based interactions, patient-centred care, team-focused care, professional responsibility and commitment (Khakbazan et al. 2019:6).

Another theme was participants’ experiences of being victimised. Women with mobility disabilities reported that the midwives emotionally and physically abused them, causing them significant difficulty, and they were afraid throughout their pregnancy and the labour process. Bassoumah and Mohammed (2020:5) reiterate that healthcare providers often expose women with disabilities to discrimination, maltreatment and humiliation through abusive and defamatory language. A study by Nguyen et al. (2019:193) also reported that women with physical disabilities experienced healthcare providers looking down on them, having negative attitudes and discriminating against them, as if they were incapable of getting pregnant. These midwives discouraged them from having children and were advising them to be sterilised. Congruently, Devkota et al. (2017:10) discovered that many women with disabilities reported negative attitudes related to healthcare providers, who often discouraged and cast doubt on these women’s sexual and reproductive health choices.

Regarding the theme of a lack of support during pregnancy, labour and puerperium, women with mobility disabilities experienced a lack of support from midwives. Studies have found that women’s satisfaction with delivery is linked to midwives showing concern and providing a conducive environment to enhance the positive effects of the childbirth process through client-centred care (Ebu, Owusu & Gross 2015:77). However, echoing this study’s findings of a lack of support from midwives, Redshaw et al. (2013:4) highlight that a proportion of women with disabilities reported they were sometimes denied the assistance they required when they contacted a midwife.

Several studies concur with the theme of participants experiencing challenges before and after giving birth related to a lack of protocols, equipment and infrastructure. In their study, Nguyen et al. (2019:192) reported on the long queues for women with physical disabilities. Furthermore, Ganle et al. (2016:8) found that information and advice from healthcare providers were sometimes irrelevant or not applicable to women with disabilities.

Women with mobility disabilities reported they experienced transport challenges when attempting to access maternal health facilities. Ganle et al. (2016:6) support this finding, as their research revealed similar mobility challenges, including an unfriendly public transport system for women with physical disabilities. Nguyen et al. (2019:191) also reiterate an inaccessible, insufficiently equipped public transport system for women with physical disabilities. In addition, Sonalkar et al. (2020:138) reported that women with physical disabilities emphasised the importance of addressing transportation issues related to health facility visits.

The study’s findings allude to a lack of infrastructure and equipment as deterrents to quality maternity care for women with mobility disabilities. This finding was supported by Devkota et al. (2017:13) and Ganle et al. (2016:8), who in their studies revealed that women with physical disabilities reported most healthcare facilities lack appropriate infrastructure such as ramps and separate and accessible toilets for persons with disabilities. It has also been reported that some women with mobility disabilities on crutches lose their balance and fall on slippery floors, as was the case in the current study. Iezzoni et al. (2015:1008) also echoed that women with mobility disabilities could not get onto their obstetrician’s fixed-height examination tables; they were thus examined while sitting in their wheelchairs or not examined at all, posing a challenge late in their pregnancy. Furthermore, Mitra et al. (2016:459) state that a lack of infrastructure in the form of inaccessible medical offices, hospital rooms, toilets, examination tables and hospital beds was a challenge for women with disabilities.

The report of participants experiencing the need for support from trained midwives is also substantiated in other research studies. According to Devkota et al. (2017:7), women with physical disabilities reported not being given complete antenatal care check-ups or counselling when using maternity services; some procedures were omitted owing to their disability. In addition, Mitra et al. (2016:459) found that many women with physical disabilities in their study described a real lack of information within the obstetrician-gynaecologist community about the effect of their disability on pregnancy, causing women with disabilities to rely on their own research and information from other women with physical disabilities. Ganle et al. (2016:8), Hall et al. (2018:5), Smeltzer et al. (2016:783) and Devkota et al. (2018:16) claim that healthcare providers appeared ill-prepared to address the maternity needs of women with disabilities. There is thus a need for appropriate training, so healthcare providers will be knowledgeable and skilled in providing adequate and quality care to women with mobility disabilities.

### Strengths and limitations

The research topic had never been explored in Eswatini, so there was a clear gap that needed to be addressed regarding women with mobility disabilities’ maternity care. Adhering to the stringent protocols of the research methodology and design, receiving continuous supervision from professors from the University of Johannesburg and the study’s evaluation by an external examiner ensured the trustworthiness of the study’s results.

The study was limited to two of the four regions of Eswatini, namely Hhohho and Manzini. The study also only focused on mobility disabilities, and future research could be conducted to cover all other forms of disabilities.

### Implications and recommendations

It is evident from the findings that there was a need for women with mobility disabilities’ maternity care to be prioritised in maternal practice. Guidelines for the implementation of holistic maternity care are needed to facilitate support and holistic maternity care for women with mobility disabilities. These could include empowering midwives to practise professionalism, respectful communication and compassion in caring for these women. There is also a need to formulate protocols and design equipment and infrastructure specifically tailored towards promoting optimal health for women with mobility disabilities. The study’s findings also reflect the need to train midwives and other health professionals to provide competent maternity care for women with mobility disabilities during pregnancy, labour and puerperium.

## Conclusion

Women with mobility disabilities experienced challenges during pregnancy, labour and puerperium. Thus, it is important that guidelines be formulated to facilitate support and holistic maternity care for women with mobility disabilities. Stakeholders of healthcare facilities need to engage in prioritising maternity care for women with mobility disabilities.
